# Program Use and Outcome Change in a Web-Based Trauma Intervention: Individual and Social Factors

**DOI:** 10.2196/jmir.5839

**Published:** 2016-09-09

**Authors:** Zhiyun Wang, Jianping Wang, Andreas Maercker

**Affiliations:** ^1^School of PhilosophyDepartment of PsychologyWuhan UniversityWuhanChina; ^2^Department of PsychologyUniversity of ZurichZurichSwitzerland; ^3^School of PsychologyBeijing Normal UniversityBeijingChina

**Keywords:** Web-based intervention, program use, trauma, adherence, social support

## Abstract

**Background:**

Insight into user adherence to Web-based intervention programs and into its relationship to intervention effect is needed.

**Objective:**

The objective of this study was to examine use of a Web-based self-help intervention program, the Chinese version of My Trauma Recovery (CMTR), among Chinese traumatized individuals, and to investigate the relationship between program use and user characteristics before the intervention and change in outcomes after the intervention and at 3-months’ follow-up.

**Methods:**

The sample consisted of 56 urban survivors of different trauma types and 90 rural survivors of the 2008 Sichuan earthquake, who used the CMTR in 1 month on their own or guided by volunteers in a counseling center. Predictors were demographics (sex, age, highest education, marital status, and annual family income), health problems (trauma duration, posttraumatic symptoms, and depression), psychological factors (coping self-efficacy), and social factors (social functioning impairment and social support). Program use was assessed by general program usage (eg, number of visiting days) and program adherence (eg, webpages completed in modules). Outcome measures were the Posttraumatic Diagnostic Scale (PDS), Symptom Checklist 90-Depression (SCL-D), Trauma Coping Self-Efficacy scale (CSE), Crisis Support Scale (CSS), and Social Functioning Impairment questionnaire (SFI) adopted from the CMTR.

**Results:**

(1) Program use: rural participants had a larger total number of visiting days (F_1,144_=40.50, *P*<.001) and visited more program modules in 1 month (χ^2^_3_=73.67, *P*<.001) than urban participants. (2) Predictors and program use: total number of visiting days was correlated with CSS at pretest (*r*=.22, *P*=.009), and total number of completed webpages was associated with SFI at pretest (*r*=.19, *P*=.02). Number of webpages completed in modules was correlated with all demographic, disease severity, psychological, and social factors at pretest. (3) Program use and outcomes change: in general, use of the triggers and self-talk modules showed a consistent positive association with improvement in PDS, SCL-D, SFI, and CSE. The relaxation module was associated with positive change in PDS, but with negative change in CSS and SFI. The professional help module was associated with positive change in SCL-D, but its use on the first day was associated with negative change in CSS and CSE. The unhelpful coping module was associated with negative change in SFI. The mastery tools module showed a consistent association with negative change in PDS and SCL-D.

**Conclusions:**

These findings suggest that both individual (eg, demographic, health problems, psychological) and social factors (eg, social functioning, social support) should be considered when delivering Web-based interventions, particularly in collectivist cultures. Specific program adherence indicators (eg, webpages completed in each module, activity types completed), rather than general program usage indicators (eg, total number or time of visiting), should be developed to examine the effectiveness of various program modules or elements.

**Clinical Trial:**

Australian New Zealand Clinical Trials Registry: ACTRN12611000951954; https://www.anzctr.org.au/Trial/Registration/TrialReview.aspx?id=343399 (Archived by WebCite at http://www.webcitation.org/6G7WyNODk)

## Introduction

In recent years, the Internet has been adopted as a valuable tool to deliver physical and mental health services to large populations [1]. Research has revealed significant treatment effects of Web-based intervention programs for a variety of mental disorders, such as depression, anxiety, and posttraumatic stress disorder (PTSD) [2,3]. However, increasing the effectiveness of Web-based intervention programs faces challenges, such as high dropout and poor user adherence reported in previous studies [4,5]. According to Christensen and colleagues [6], dropout refers to a participant not completing the research trial protocol or trial assessments associated with a Web-based intervention; and adherence refers to the extent to which participants experience the content of the Web-based intervention, which is the focus of this study. Further, adherence can be examined by the indicators of general program usage (eg, number of log-ins or time spent on intervention programs) and program adherence (ie, the extent to which intervention programs are used in accordance to recommendations, such as content modules completed or activities completed) [7].

The relationship between user exposure to intervention programs and the effect of Web-based interventions can be complicated [8] and may be a dose-response relationship [9]. Donkin et al [10] found, however, that compared with low program users, medium users showed little additional benefit from Web-based intervention, and suggested that “concentrated use of the program (eg, completing multiple modules per log-in) or passive exposure to material (as measured by modules completed) may not be as useful as regular shorter periods of use with higher levels of activity in each of these log-ins.” Thus, deeper insight into the process of Web-based program use and into its relationship to intervention effect is needed.

To improve Web-based interventions for target groups with various physical and mental health problems, previous studies have investigated potential predictors of program use, with mixed findings. One group of predictors is user characteristics, including (1) demographic variables, such as age, sex, education, marital status, and socioeconomic status [7,11], (2) health problems, such as severity of the target disease, duration of the target disease, and subjective health status [12,13], (3) psychological factors, such as illness attitudes and beliefs, expectations, motivation, and self-efficacy [6,14], and (4) social factors, such as family characteristics (eg, parenting practices and styles, socioeconomic status of the family), and support from partners and friends [12,15]. Another group of predictors includes the characteristics of Web-based interventions, such as feedback, interactive elements, email or telephone contact, and reminders [16,17].

Based on these findings, this study investigated the role of demographics, health problems, and psychological and social factors in Chinese traumatized persons’ use of a Web-based self-guided intervention program, the Chinese version of My Trauma Recovery (CMTR). The program showed preliminary short-term treatment effects on PTSD and depressive symptoms in a randomized controlled trial in 2 Chinese populations [18]. The trial showed a high dropout rate, however, with 40.8% of participants not completing the research protocol [19-21]. Further analysis revealed that participant dropout was associated with such social factors as needs in trauma disclosure and perceived social acknowledgment or disapproval by family and extended social environments. This indicated a necessity to examine social factors together with other factors for better understanding of program use across cultures.

Note that, although it has been argued that Web-based interventions are potentially beneficial for rural residents to receive mental health help, because they have little access to face-to-face mental health resources [22], few empirical studies have evaluated the use and efficacy of such programs among rural users. More precisely, previous studies primarily focused on Internet users, who might come from rural as well as urban areas, and found that more highly educated, older, and female users were more likely to adhere to Web-based interventions [23]. However, for this study we recruited non-Internet users from rural areas who met the required literacy level for Web-based interventions. These people tend to be older and less educated, to have a lower income, and typically have much less Internet use experience. Volunteers provided them with (minimal) support with Internet service problems in a counseling center so that they could complete and benefit from the CMTR program [18]. The literature suggests that social support and contact would increase the use of Web-based intervention programs, particularly the number and duration of visits [16]. This study thus examined the difference in program use between rural and urban users. It offers implications for future application of Web-based interventions, for example, in psychological and mental health aid for extensive rural populations who are in need of mental health service but have little access even to online mental health resources.

In sum, this study aimed to investigate (1) how urban and rural participants used the CMTR program, including general program usage and program adherence, (2) how program use was related to demographics (ie, sex, age, highest level of education attained, marital status, and annual family income), health problems (ie, PTSD and depressive symptom severity, trauma duration), psychological factors (ie, coping self-efficacy), and social factors (ie, social functioning impairment and social support after trauma) before the intervention, and (3) how program use was associated with change in outcomes after the treatment and at 3-months’ follow-up.

## Methods

### Materials

The CMTR program was translated, with minimal cultural adaptation of pictures, audio and video segments, and professional hotlines, from the English My Trauma Recovery program (previously referred to as Journey to Trauma Recovery), which aims to improve trauma recovery by increasing individual coping self-efficacy and coping skills [24]. CMTR contains 6 recovery modules offering education and exercises for 6 trauma recovery-related topics: professional help, relaxation, self-talk, social support, triggers, and unhelpful coping [24]. There are 2 other interactive sections: self-test and mastery tools. Specifically, users are encouraged to take the self-test on posttraumatic stress reactions and coping self-efficacy after log-in and once a week during the treatment period. The mastery tools section offers hyperlinks to all exercises in the 6 recovery modules, so that users can easily access them in future. Because few users took the self-test during the treatment period, we report only the data on the 6 recovery modules and the exercise section.

### Sample and Procedure

We used a sample of 56 urban and 90 rural participants, which was part of the sample (ie, 90 urban and 93 rural participants who completed the pretest) reported earlier [18]. Specifically, of the 93 rural participants, we excluded 3 for not using the 6 recovery modules and the mastery tools section after log-in. Of the 90 urban participants, we excluded 29 for not logging in and 5 for not using the 7 modules after log-in. Based on chi-square analysis results, the 34 urban dropouts (ie, 29 plus 5) did not differ significantly from the 56 participants in sex, age, marital status, highest education level attained, and family income. Analyses of variance (ANOVAs) revealed significant difference in the Crisis Support Scale (CSS) (dropouts: mean score 1.93, SD 0.85; users: mean score 1.58, SD 0.72; *F*_1,88_=4.23, *P*=.04), but no significant differences in the Posttraumatic Diagnostic Scale (PDS), Symptom Checklist 90-Depression (SCL-D), Social Functioning Impairment questionnaire (SFI), or Trauma Coping Self-Efficacy scale (CSE) (*F*_1,88_ range 0.11–1.28, all *P*>.26) at pretest.

Urban and rural participants used the program in some different way. The urban participants had experienced a variety of traumatic events (eg, physical assault, unexpected death of someone close, or serious accidents), reported at least two PTSD symptoms in a trauma screening questionnaire, and had sufficient Internet access time (≥360 minutes in 4 weeks). They had Internet access at home or work and thus decided themselves when, where, and how often to visit the program during the 1-month treatment period. Research assistants did not contact them or give reminders until posttest. The rural participants were survivors of the 2008 earthquake in Beichuan county in Sichuan province, and reported at least two PTSD symptoms in the trauma screening questionnaire. Due to lack of personal Internet access, they used the program 5 times (at least 30 minutes per time) in a counseling center’s computer room. Some participants used the program more than 5 times because they broke off one or more intervention sessions for personal reasons and then made them up later. Note that research assistants provided support for all urban and rural participants only for technical problems with CMTR. When participants asked for support with their distress or the program content, research assistants first evaluated the participants’ needs and, if these were not acute, sent the participants a brief reply that CMTR was a self-help program and they would get further support, if needed, after the follow-up test. Otherwise, the research assistants would interrupt the session for other treatment, which did not happen in this study.

### Measures

#### Posttraumatic Diagnostic Scale

This scale includes 17 PTSD symptom items assessing the frequency of trauma-related symptoms in the past month on a 4-point scale (0=not at all or only 1 time, 3=5 or more times a week or almost always) [25]. The internal consistency of the scale in this study was alpha=.92.

#### Symptom Checklist 90-Depression

We used the 13-item depression subscale of the Symptom Checklist-90 [26] to measure to what extent participants had been bothered by depressive symptoms in the past month on a 5-point scale, ranging from 0 (not at all) to 4 (extremely). The internal consistency of the scale in this study was alpha=.94.

#### Social Functioning Impairment

We adopted 4 questions from the My Trauma Recovery program to examine individual functional impairment after trauma experiences. An example question is “To what extent have your reactions to what has happened reduced your ability to complete your normal responsibilities (eg, job, school, home, childcare duties)?” Participants answered the questions on a 5-point scale (0=not at all, 4=extremely). The internal consistency of the questionnaire in this study was alpha=.88.

#### Crisis Support Scale

This 7-item scale measures received practical support, received sympathy, being able to find someone to talk to about the traumatic experience, and overall satisfaction with received social support after trauma [27]. Example statements are “I can talk to someone who has had a similar experience” and “People are being supportive and empathetic of me.” Participants responded to the statements on a 5-point scale (0=not at all, 4=extremely). The internal consistency of the scale in this study was alpha=.86.

#### Trauma Coping Self-Efficacy Scale

This 10-item scale is a short version of the Coping Self-Efficacy Scale for Trauma [28]. It measures to what extent participants felt capable of coping with PTSD reactions at different assessment points. The 5-point scale ranges from 0 (not at all) to 4 (extremely). The internal consistency of the scale in this study was alpha=.83.

#### Program Use

##### Total Number of Days Visiting CMTR

After the first log-in, most participants left CMTR without logging out and then visited the program without logging in next time. It was thus difficult to calculate the number of log-ins or the amount of time spent on CMTR. Thus, we counted as 1 day when a participant visited the program within one 24-hour day. The maximum could be 30 days during the 1-month treatment period.

##### Total Number of CMTR Webpages Completed

We tracked how many CMTR webpages participants completed during the 1-month treatment period. Because participants were encouraged to use the program as many times as they wanted, webpages were counted repeatedly when repeated use occurred. Thus, the total number of webpages completed could be larger than the number of webpages contained in CMTR.

##### Number of Modules Visited

The maximum could be 7 modules in this study; that is, 6 recovery modules and the mastery tools module. Note that participants could have visited 1 module but not have completed all the webpages in the module.

##### Number and Proportion of Webpages Completed in Each Module

We calculated the proportion of each module completed during the treatment period. The number of webpages completed could be larger than the actual total number of webpages in each module due to repeated use.

##### Number of Modules Visited per Day

We tracked how many modules participants visited on different days through the treatment course. The maximum could be 7 modules.

##### Number and Proportion of Webpages Completed in Each Module on the First Day

We tracked the proportion of each module that was completed on the first day. The number of webpages completed in each module could be larger than the actual total number of webpages due to repeated use.

### Data Analysis

We analyzed the data using IBM SPSS version 22 (IBM Corporation). Chi-square analysis tested urban and rural subsample differences in demographic characteristics. We used 1-way ANOVA to test subsample differences in the pretest scores on PDS, SCL-D, SFI, CSS, and CSE, and in program use indicators. We conducted 1-way ANOVA and correlation analyses to explore the relationship between program use and demographics, health problems, psychological factors, and social factors at pretest. Finally, we conducted linear regression analysis for PDS, SCL-D, SFI, CSS, and CSE posttreatment incremental difference scores (ie, posttest values minus pretest values, then divided by the variance of pretest values). In the linear regression analysis, we entered trauma duration and 6 dummy variables as independent variables in the first block (stepwise method; these were sex female or not, age 26–40 years old or not, family income US $0–4000 or not, marital status of married or not, highest education high middle school/bachelor’s degree or not, and sample urban or not) and the indicators of program use in the second block (enter method). Similarly, we conducted linear regression analysis for PDS, SCL-D, SFI, CSS, and CSE 3-month follow-up incremental difference scores (ie, follow-up values minus pretest values, then divided by the variance of pretest values).

## Results

### Overall Program Use

Table 1 presents the demographic statistics of the 56 urban (38.4%) and 90 rural participants (61.6%). The rural subsample consisted of more female, older, and married participants than did the urban subsample. The rural subsample also reported a lower annual family income and level of education. Table 2 shows subsample means (SD) and correlations of variables at pretest. The urban participants reported higher levels of depression and social functioning impairment, but a lower level of social support, than the rural participants. In addition, the rural participants had a longer trauma duration than the urban participants. Correlation analysis showed that a longer trauma duration was associated with participants’ lower level of depression. While PTSD symptoms, depression, and social functioning impairment correlated positively with each other, social support correlated negatively with depression and social functioning and positively with coping self-efficacy.

**Table 1:** Demographic characteristics of participants in the Chinese version of My Trauma Recovery intervention (N=146; urban: n=56; rural: n=90).

Characteristic		Urban n (%)	Rural n (%)	χ^2^	*df*	*P* value
**Sex**						
	Female	38 (67.9)	74 (82.2)	3.99	1	.046
	Male	18 (32.1)	16 (17.8)			
**Age range (years)**						
	16–25	29 (51.8)	1 (1.1)	63.98	2	<.001
	26–40	23 (41.1)	41 (45.6)			
	41–70	4 (7.1)	48 (53.3)			
**Annual family income ($US)**						
	0–4000	17 (30.4)	81 (90)	54.94	2	<.001
	4001–10,000	22 (39.3)	5 (5.6)			
	≥10,001	13 (23.2)	2 (2.2)			
	Missing	4 (7.1)	2 (2.2)			
**Marital status**						
	Single	43 (76.8)	5 (5.6)	79.37	1	<.001
	Married	13 (23.2)	85 (94.4)			
**Education**						
	Junior middle school/lower	1 (1.8)	64 (71.1)	94.36	2	<.001
	High middle school	7 (12.5)	19 (21.1)			
	Bachelor’s degree/higher	48 (85.7)	7 (7.8)			

**Table 2:** Subsample difference and correlations at pretest in the Chinese version of My Trauma Recovery intervention (N=146; urban: n=56; rural: n=90).

Test	Subsample scores, mean (SD)		*F*_1,144_	*P* value	PDS^a^	*P* value	SCL-D^b^	*P* value	SFI^c^	*P* value	CSS^d^	*P* value	CSE^e^	*P* value
	Urban	Rural												
PDS	1.70 (0.57)	1.70 (0.51)	0.00	.99	1									
SCL-D	2.53 (0.83)	2.13 (0.81)	7.91	.006	.68	<.001	1							
SFI	2.65 (0.95)	2.11 (0.96)	10.96	.001	.64	<.001	.62	<.001	1					
CSS	1.63 (0.75)	2.26 (0.55)	34.40	<.001	–.13	.12	–.31	<.001	–.24	.004	1			
CSE	1.99 (0.67)	1.91 (0.53)	0.75	.39	–.08	.31	–.06	.50	.04	.62	.32	<.001	1	
DUR^f^	30.16 (45.71)	49.66 (4.24)	16.22	<.001	–.14	.09	–.21	.01	–.06	.45	.15	.07	–.06	.47

^a^PDS: Posttraumatic Diagnostic Scale.

^b^SCL-D: Symptom Checklist 90-Depression scale.

^c^SFI: Social Functioning Impairment.

^d^CSS: Crisis Support Scale.

^e^CSE: Trauma Coping Self-Efficacy scale.

^f^DUR: Trauma duration (in months).

On average, the total number of days visiting CMTR was 3.50 (SD 2.82; minimum 1, maximum 12) among urban participants, of whom 80% (45/56) visited the modules for ≤4 days. The total number of days was 5.51 (SD 0.81; minimum 4, maximum 8) among rural participants, of whom 87% (78/90) used the modules for 5 or 6 days. The subsample difference reached significance (*F*_1,144_=40.50, *P*<.001). Urban and rural participants did not differ in the total number of CMTR webpages completed (urban: mean 88.05, SD 76.86; rural: mean 100.13, SD 14.14; *F*_1,144_=2.12, *P*=.15).

### Module Use

On average, the number of modules visited was 4.63 (SD 2.17) for urban participants and 5.48 (SD 0.66) for rural participants (see Table 3). Chi-square analysis, with categories 1 to 4 combined, revealed a significant subsample difference (χ^2^_3_=73.67, *P*<.001).

Table 4 shows the number of webpages completed in each module during the treatment period (top half). The rural participants completed significantly more webpages in the relaxation, unhelpful coping, professional help, and social support modules, but fewer webpages in the trauma triggers and mastery tools modules than the urban participants.

**Table 3:** Number of modules visited among urban and rural participants in the Chinese version of My Trauma Recovery intervention.

Number of modules	Urban (n=56)		Rural (n=90)	
	Frequency	Proportion	Frequency	Proportion
1	4	7.1%		
2	10	17.9%		
3	7	12.5%		
4	4	7.1%	3	3.3%
5	7	12.5%	46	51.1%
6	5	8.9%	36	40.0%
7	19	33.9%	5	5.6%

**Table 4:** Number and proportion of completed webpages in each module in the Chinese version of My Trauma Recovery intervention.

Module^a^		Urban (n=56)			Rural (n=90)			*F*_1,144_	*P* value
		Mean	SD	%	Mean	SD	%		
**In 1 month**									
	Relaxation	4.18	4.99	104.5	6.02	1.69	150.6	10.40	.002
	Triggers	7.41	9.80	74.1	2.70	6.19	27.0	12.69	<.001
	Coping	8.86	11.83	55.4	17.62	6.79	110.1	32.38	<.001
	Help	4.34	6.57	62.0	10.84	4.47	154.9	50.68	<.001
	Self-talk	24.34	23.10	93.6	27.42	4.33	105.5	1.52	.22
	Support	13.13	14.69	77.2	17.89	7.64	105.2	6.61	.01
	Tools	4.86	4.97	69.4	0.60	1.26	8.6	60.12	<.001
**On the first day**									
	Relaxation	1.23	1.94	30.8	1.58	2.74	39.4	0.68	.41
	Triggers	1.38	4.30	13.8	0.23	1.48	2.3	5.35	.02
	Coping	2.29	5.15	14.3	0.29	2.74	1.8	9.32	.003
	Help	0.88	2.85	12.5	3.78	4.65	54.0	17.65	<.001
	Self-talk	7.64	14.12	29.4	3.06	8.75	11.8	5.88	.02
	Support	4.70	8.15	27.6	1.90	5.74	11.2	5.90	.02
	Tools	1.84	2.43	26.3	0.19	0.93	2.7	33.57	<.001

^a^The total number of webpages in the 7 modules (from top to bottom) is 4, 10, 16, 7, 26, 17 and 7.

### Program Use per Day

**Figure 1:**
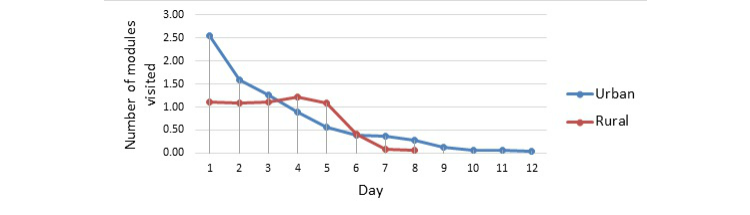
Number of modules visited per day by urban (n=56) and rural (n=90) participants in the Chinese version of My Trauma Recovery intervention.

As Figure 1 shows, the number of modules visited by urban participants decreased through the treatment course, while rural participants’ module use was relatively stable. Table 4 presents the number of webpages completed in each module on the first day (bottom half). While the rural participants completed significantly more webpages in the professional help module than the urban participants, the urban participants completed more webpages in other modules except the relaxation module on the first day.

### Program Use and User Characteristics at Pretest

A 1-way ANOVA among urban and rural participants revealed no significant sex, age, education, marital status, and family income differences in the total number of visiting days or the total number of CMTR webpages completed (all *P*>.12). As for the number of completed webpages in each module, the urban participants aged 26–40 years completed significantly more webpages in the mastery tools module than those aged 16–25 years (*F*_2,53_=3.39, *P*=.04). Among rural participants, female participants visited more webpages in the professional help module than did male participants (*F*_1,88_=5.02, *P*=.03), and married participants visited more webpages in the relaxation module (*F*_1,88_=5.11, *P*=.03). In addition, correlation analysis showed no significant relationship between trauma duration and the 3 program use indicators in each subsample.

Table 5 presents the correlations between PDS, SCL-D, SFI, CSS, and CSE pretest scores and the program use indicators. The total number of days was positively correlated with CSS score, and total number of webpages completed was positively correlated with SFI score. The number of webpages completed in the modules during the treatment period showed a positive association with PDS, SCL-D, SFI, and CSE scores, but a negative correlation with the CSS score. In general, these variables showed more significant correlations with the number of webpages completed in the modules on the first day than the number completed in 1 month.

### Program Use and Outcomes Change at Posttest and Follow-Up

Table 6 shows the results of regression analysis of PDS, SCL-D, SFI, CSS, and CSE posttreatment and follow-up incremental difference scores on the number of webpages completed in each module in 1 month. Table 7 showed the results of regression analysis when the number of webpages completed in each module on the first day was entered in the second block as an independent variable.

**Table 5:** Correlations of program use and variable scores at pretest in the Chinese version of My Trauma Recovery intervention (N=146).

Program use indicator		PDS^a^	*P* value	SCL-D^b^	*P* value	SFI^c^	*P* value	CSS^d^	*P* value	CSE^e^	*P* value
Total number of days		–.04	.65	–.06	.47	.02	.82	.22	.009	.06	.51
Total number of webpages completed		.11	.19	.08	.33	.19	.02	–.03	.76	.13	.11
**Number of pages completed in each module in 1 month**																	
	Relaxation	.15	.07	.11	.19	.11	.18	.10	.25	.13	.11
	Triggers	.04	.62	.15	.08	.18	.03	–.07	.38	.07	.43
	Coping	.08	.37	–.09	.29	–.01	.92	.12	.15	.05	.53
	Help	.20	.02	.10	.23	.18	.03	.15	.08	.04	.66
	Self-talk	.08	.33	.03	.72	.15	.08	.01	.89	.06	.48
	Support	.03	.69	.03	.71	.07	.39	–.07	.39	.16	.06
	Tools	.10	.24	.20	.02	.32	<.001	–.29	<.001	.17	.04
**Number of pages completed in each module on the first day**																	
	Relaxation	.02	.78	–.03	.69	–.02	.86	.13	.11	.01	.86
	Triggers	.18	.03	.25	.002	.24	.004	–.13	.13	–.05	.55
	Coping	.19	.02	.23	.006	.20	.02	–.21	.01	–.01	.92
	Help	.15	.08	.06	.51	–.01	.86	.22	.009	.05	.55
	Self-talk	.08	.33	.17	.04	.15	.07	–.22	.006	–.23	.006
	Support	.15	.07	.23	.005	.12	.16	–.19	.02	.07	.38
	Tools	.15	.08	.24	.004	.22	.009	–.29	<.001	.02	.81

^a^PDS: Posttraumatic Diagnostic Scale.

^b^SCL-D: Symptom Checklist 90-Depression.

^c^SFI: Social Functioning Impairment.

^d^CSS: Crisis Support Scale.

^e^CSE: Trauma Coping Self-Efficacy scale.

**Table 6:** Regressions on the number of webpages completed in each module in 1 month by participants in the Chinese version of My Trauma Recovery intervention (N=146).

Module		PDS^a^			SCL-D^b^			SFI^c^			CSS^d^			CSE^e^		
		b	Beta	*P* value	b	Beta	*P* value	b	Beta	*P* value	b	Beta	*P* value	b	Beta	*P* value
**Posttreatment incremental difference (n=122)**																
Relaxation		–0.01	–0.02	.89	–0.03	–0.08	.47	1.34	0.22	.04	–0.01	–0.04	.75	–0.06	–0.16	.17
Triggers		0.01	0.04	.71	0.01	0.04	.71	–0.16	–0.06	.55	–0.01	–0.05	.67	0.01	0.07	.55
Coping		0.01	0.10	.40	0.02	0.14	.22	0.50	0.23	.04	0.00	0.002	.98	–0.02	–0.13	.29
Help		–0.01	–0.05	.67	–0.05	–0.25	.04	0.11	0.03	.78	–0.003	–0.02	.90	0.03	0.14	.26
Self-talk		–0.02	–0.26	.047	–0.02	–0.23	.06	–0.48	–0.33	.005	0.01	0.08	.54	0.01	0.13	.31
Support		0.01	0.10	.39	–0.01	–0.11	.31	0.01	0.004	.97	0.00	–0.003	.98	–0.00	–0.04	.73
Tools		–0.02	–0.05	.65	0.09	0.28	.046	0.001	0.00	.999	0.04	0.10	.50	–0.03	–0.08	.60
*R*^2^		.05			.17			.21			.09			.15		
**Follow-up incremental difference (n=113)**																
Relaxation		–0.04	–0.10	.38	–0.02	–0.05	.62	1.35	0.17	.09	–0.08	–0.21	.07	–0.07	–0.16	.19
Triggers		0.02	0.12	.28	0.02	0.10	.35	0.18	0.05	.58	–0.01	–0.03	.78	0.05	0.24	.04
Coping		–0.01	–0.09	.48	0.00	0.03	.79	0.57	0.21	.049	–0.01	–0.05	.69	–0.01	–0.04	.75
Help		0.03	0.13	.32	–0.01	–0.06	.63	–0.42	–0.10	.37	–0.01	–0.05	.71	0.00	0.003	.98
Self-talk		–0.01	–0.07	.59	–0.02	–0.17	.15	–0.79	–0.39	<.001	0.01	0.13	.29	0.01	0.08	.52
Support		–0.01	–0.10	.39	–0.01	–0.05	.63	–0.22	–0.09	.36	0.01	0.10	.36	–0.01	–0.07	.54
Tools		0.08	0.26	.07	0.10	0.31	.02	0.17	0.02	.81	–0.03	–0.08	.55	–0.02	–0.06	.61
*R*^2^		.17			.26			.28			.27			.07		

^a^PDS: Posttraumatic Diagnostic Scale.

^b^SCL-D: Symptom Checklist 90-Depression.

^c^SFI: Social Functioning Impairment.

^d^CSS: Crisis Support Scale.

^e^CSE: Trauma Coping Self-Efficacy scale.

**Table 7:** Regressions on the number of webpages completed in each module on the first day by participants in the Chinese version of My Trauma Recovery intervention (N=146).

Module		PDS^a^			SCL-D^b^			SFI^c^			CSS^d^			CSE^e^		
		b	Beta	*P* value	b	Beta	*P* value	b	Beta	*P* value	b	Beta	*P* value	b	Beta	*P* value
**Posttreatment incremental difference (n=122)**																
Relaxation		–0.06	–0.13	.18	0.00	0.01	.93	0.58	0.07	.42	–0.10	–0.20	.04	–0.02	–0.03	.73
Triggers		–0.04	–0.10	.49	–0.04	–0.10	.46	–1.53	–0.21	.07	–0.00	–0.01	.97	0.04	0.09	.47
Coping		0.01	0.04	.77	–0.001	–0.002	.99	1.35	0.27	.014	0.03	0.10	.41	–0.01	–0.04	.76
Help		–0.02	–0.09	.36	–0.05	–0.23	.03	–0.04	–0.01	.93	–0.06	–0.23	.03	–0.05	–0.17	.09
Self-talk		–0.01	–0.05	.62	–0.02	–0.17	.09	–0.33	–0.18	.06	0.01	0.06	.56	0.02	0.21	.04
Support		–0.00	–0.01	.93	–0.01	–0.05	.63	0.11	0.04	.71	–0.02	–0.12	.26	–0.01	–0.06	.54
Tools		–0.02	–0.02	.86	0.09	0.13	.28	1.92	0.16	.15	0.04	0.05	.71	–0.11	–0.15	.21
*R*^2^		.04			.15			.22			.14			.18		
**Follow-up incremental difference (n=113)**																
Relaxation		–0.10	–0.21	.06	–0.03	–0.07	.50	1.22	0.12	.21	–0.07	–0.16	.12	–0.08	–0.14	.19
Triggers		0.04	0.04	.68	0.07	0.08	.35	–0.10	–0.01	.95	–0.08	–0.09	.31	–0.05	–0.04	.65
Coping		0.02	0.05	.58	–0.02	–0.04	.68	1.22	0.16	.08	0.01	0.01	.89	0.01	0.02	.88
Help		–0.04	–0.16	.18	–0.01	–0.03	.76	0.24	0.04	.69	–0.04	–0.14	.21	–0.07	–0.21	.045
Self-talk		–0.01	–0.11	.28	–0.02	–0.18	.06	–0.08	–0.03	.74	0.01	0.11	.24	0.02	0.14	.17
Support		–0.01	–0.04	.70	–0.02	–0.09	.36	0.07	0.01	.89	0.02	0.11	.27	0.01	0.04	.73
Tools		0.09	0.11	.37	0.07	0.09	.45	0.47	0.03	.78	–0.10	–0.12	.29	0.01	0.01	.91
*R*^2^		.15			.23			.19			.28			.08		

^a^PDS: Post-traumatic Diagnostic Scale.

^b^SCL-D: Symptom Checklist 90-Depression.

^c^SFI: Social Functioning Impairment.

^d^CSS: Crisis Support Scale.

^e^CSE: Trauma Coping Self-Efficacy scale.

As shown, after controlling for demographic variables, subsample, and trauma duration, use of the relaxation (on the first day), professional help (in 1 month, on the first day), and self-talk (in 1 month, on the first day) modules was associated with score reductions in the PDS and SCL-D. However, use of the mastery tools module in 1 month was unexpectedly associated with score increases in PDS and SCL-D.

In addition, use of the triggers (in 1 month) and self-talk (on the first day) modules was associated with score increases in CSE, but use of the professional help module on the first day was associated with a score reduction in CSE.

As for social variables, use of the triggers (on the first day) and self-talk (in 1 month, on the first day) modules was associated with score reductions in SFI. However, use of the relaxation (in 1 month) and unhelpful coping (in 1 month, on the first day) modules was associated with score increases in SFI. Also, use of the relaxation (in 1 month, on the first day) and professional help (on the first day) modules was associated with score reductions in CSS.

## Discussion

### Main Findings

#### Program Use

The urban and rural participants used the CMTR program for 1 month in different ways and showed different patterns of program use. In general, the rural participants used the program for more days, visited more modules, and completed more webpages in most modules than the urban participants in 1 month. However, the rural participants may actually have been passive users compared with the urban participants. First, they completed a much smaller proportion of the mastery tools module, which contains all of the tools and skills exercises. Second, while the urban participants quickly decreased the number of modules they visited from the second day, which is consistent with previous findings [29], the rural participants visited on average 1 module in each session. This might have been due to the prescribed intervention procedure among these rural users. In this sense, the program adherence indicator of number of webpages completed in each module is more informative than other general program use indicators.

It is important to note that the relatively passive program use among rural participants might have been due to their lack of Internet use experience (eg, being less skilled at module switching) rather than lack of motivation. The data revealed that the rural participants completed a larger proportion of the professional help module on the first day, as well as during the treatment period, which may indicate a motivation to receive interventions. If this is the case, future studies could effectively increase the program use in rural populations by improving their Internet use skills before applying interventions.

#### User Characteristics and Program Use

The total number of visiting days and completed webpages showed a positive association only with social factors. That is, users with higher social support and social functioning impairment tended to use the program more. As for the number of webpages completed in the modules, demographic, disease severity, and psychological and social factors all showed some significant correlations. Consistent with previous findings [23], the urban participants aged 26–40 years completed more webpages in the mastery tools module than those aged 16–25 years. Among rural participants, female and married participants visited more webpages in different modules than did male and single participants.

PTSD symptoms and depression before treatment were positively related to module use, particularly to that on the first day. This suggests that disease severity has a motivational role in program use, which is consistent with previous findings [6]. Moreover, the better correlation on the first day supports the CMTR program’s original intention: that users will be directed to those modules that may be most useful for them based on their self-test results. These findings thus might help us to learn about users’ needs and then improve the intervention efficacy even with limited program use. Given that a large number of individuals dropped out after a few sessions but still benefited from Web-based interventions [18,30], it may be valuable in future research to design short-term as well as long-term use patterns of such interventions in order to benefit more users.

While social functioning impairment was positively related to module use, similar to disease severity variables, our findings on the other social factor (ie, social support) were more complex. Social support at pretest was positively associated with the total number of days visiting CMTR, but negatively related to the use of most modules, except for the professional help module, which introduces what happens in face-to-face professional counseling. These findings indicated that, on the one hand, individuals with less social support might be less interested in seeking face-to-face professional help, and be motivated to use Web-based intervention programs. On the other hand, more social support might provide resources for individuals to insist on using a self-help intervention program.

The motivational role and resource role of social support were also reported in previous studies [31]. For example, Yli-Uotila et al [32] found that patients with cancer used the Internet as a source of social support due to the need for emotional and informational support, a lack of support outside the Internet, the ease of communication, and the negative experiences caused by the illness. As for the resource role, previous studies revealed that support from partners and friends was associated with individual adherence to Web-based interventions [12]. Thus, further research should pay attention to the various roles of social factors to better understand who chooses Web-based interventions and why, and how they use the program through the intervention course, particularly in collectivist cultures.

Finally, participants’ coping self-efficacy was negatively related to the use of the self-talk module on the first day, but positively related to use of the mastery tools module in 1 month. Thus, coping self-efficacy might also play a motivational role in pushing individuals to use the program content, and resources could be offered for individuals particularly to complete interactive tasks (eg, exercises and activities in a program), providing further support for the role of self-efficacy in improving Web-based intervention program use [12,13].

#### Program Use and Outcomes Change

Among the 7 modules that we examined in this study, the self-talk and triggers modules showed a consistent association with improvement in 4 outcomes (ie, PTSD symptoms, depression, coping self-efficacy, and social functioning impairment). The relaxation module was associated with positive change in PTSD symptoms, but with negative change in 2 social variables (ie, social functioning impairment and social support). The professional help module was associated with a positive change in depression, but its use on the first day was related to a negative change in coping self-efficacy and social support. The unhelpful coping module showed an association with negative change in social functioning impairment. The mastery tools module use in 1 month showed a consistent association with a negative change in disease severity variables (ie, PTSD symptoms and depression).

These findings provide a better understanding of how the CMTR program worked to help the participants in dealing with their PTSD symptoms and depression after trauma [18]. They also showed that the CMTR modules did not play a unified role in treatment. Thus, the effectiveness of various elements in Web-based intervention programs should be examined (eg, education, self-monitoring, feedback or tailored information, self-management training, and personal exercise program) [33]. Also, future studies need to examine different delivery forms of these elements, for example, providing exercises outside of or within its educational context.

Moreover, it is surprising that the CMTR modules seem to have had much less of an effect in improving users’ social support and social functioning impairment. Given that individuals turn to the Internet for social support, as well as for health information, the perceived low effectiveness in social support improvement may also cause user dropout and low adherence to Web-based intervention programs [34]. It is thus essential to consider social factors in Web-based interventions, for example, providing special social support-related content (eg, the social support module in CMTR) for social skills training, or using some form of service delivery (eg, contact with therapists or other users, forum posts, and blog posts; [35]) to offer social experiences for users. This is also one goal in future research on the CMTR program. Other goals are updating contents based on the latest research advances and making more cultural adaptations.

### Limitations

First, this study had limitation in sampling. Due to the small size and self-selected sample, our findings cannot be generalized to populations from hospitals or outpatient clinics. Future studies need to examine CMTR program use in a larger representative sample. In particular, attention should be paid to the difference in Web-based intervention program use between urban and rural users. As a potential target group of Web-based interventions, rural residents may benefit from (minimal) guided self-help interventions, although lack of Internet use experience could hinder their full use of these interventions. Future research should investigate more directly the impact of this group’s other characteristics (eg, Internet use experience, lay beliefs about health problems) on their program use and explore effective methods to improve their program use in a practical way, such as offering short-term computer training before interventions.

A second limitation was a lack of control over the impact of program factors (eg, different number of webpages in modules, and the types of webpages in modules) on module use. Such an impact might be complex. For example, the self-talk, social support, and unhelpful coping modules all contain a relatively large number of webpages (ie, 16–26 pages), but the (urban) participants used them in very different ways. Thus, more specific indicators should be adopted to assess the active use of these program elements rather than passive exposure to the general program.

Finally, the findings on first-day and 1-month program use have implications for further research. Finding out user and program characteristics that promote initial program use and continuous program use will be helpful. Understanding the quicker and slower effectiveness of various program elements would also be helpful.

## Abbreviations

ANOVA: analysis of variance
CMTR: Chinese version of My Trauma Recovery
CSE: Trauma Coping Self-Efficacy scale
CSS: Crisis Support Scale
PDS: Posttraumatic Diagnostic Scale
PTSD: posttraumatic stress disorder
SCL-D: Symptom Checklist 90-Depression
SFI: Social Functioning Impairment
